# Bridging the Holistic-Reductionist Divide in Microbial Ecology

**DOI:** 10.1128/mSystems.00265-18

**Published:** 2019-02-05

**Authors:** Robin Tecon, Sara Mitri, Davide Ciccarese, Dani Or, Jan Roelof van der Meer, David R. Johnson

**Affiliations:** a Department of Environmental Systems Science, ETH Zürich, Zürich, Switzerland; b Department of Fundamental Microbiology, University of Lausanne, Lausanne, Switzerland; c Department of Environmental Microbiology, EAWAG, Dübendorf, Switzerland

**Keywords:** metagenomics, microbial communities, microbial diversity, synthetic ecology

## Abstract

Microbial communities are inherently complex systems. To address this complexity, microbial ecologists are developing new, more elaborate laboratory models at an ever-increasing pace. These model microbial communities and habitats have opened up the exploration of new territories that lie between the simplicity and controllability of “synthetic” systems and the convolution and complexity of natural environments. Here, we discuss this classic methodological divide, we propose a conceptual perspective that integrates new research developments, and we sketch a 3-point possible roadmap to cross the divide between controllability and complexity in microbial ecology.

## Abstract (precis)

Microbial communities are inherently complex systems. To address this complexity, microbial ecologists are developing new, more elaborate laboratory models at an ever-increasing pace.

## PERSPECTIVE

Everything simple is false. Everything complex is unusable.

—Paul Valéry (*Mauvaises pensées et autres*, Gallimard, 1942)

The daunting complexity of microbial communities compels microbial ecologists into two alternative methodological corners: holistic and reductionist (1). The holistic approach posits that microbial communities are best understood and studied as a whole, where dense interaction networks and natural environments are maintained (be it a forest soil or the human gut) and where emergent properties cannot be deduced from the study of the individual components. Meta-omics studies of environmental samples are good examples of approaches with a holistic outlook (2, 3). In contrast, the reductionist approach investigates simpler microbial communities and ecosystems, for example, where a few genotypes are assembled together experimentally and propagated in the laboratory under highly controlled conditions (e.g., synthetic microbial ecology [4, 5]). A tenet of this approach is that the quantitative study of the genetics and physiology of individual genotypes as well as their pairwise interactions can reveal the building blocks of complex microbial behaviors in nature.

Reductionism and holism are not mutually exclusive. On the contrary, the two are complementary and are usually integrated in successful systems biology programs (1, 6). Nevertheless, it appears to us that the holistic-reductionist divide is still very present in the study of microbial communities, driven by the urge to specialize in one of the two methodological frameworks. These two frameworks possess their own advantages and drawbacks. While the holistic approach offers a high degree of realism and can generate hypotheses about ecological and evolutionary mechanisms underlying community behavior, inherent confounding factors may preclude conclusive validation or refuting of those hypotheses (7). Conversely, the relative simplicity and experimental control of simpler microbial communities facilitate hypothesis testing and offer some degree of prediction (under a heavy set of assumptions). However, the mechanisms identified and their ability to explain the behaviors of natural microbial communities is questionable.

Holistic and reductionist microbial ecology thus operate at opposing and extreme ends of the control-complexity spectrum, with a largely uncharted territory between the two. Can we bridge this divide? Will this help to achieve a better understanding of microbial community formation, structure, functioning, and evolution? Similar questions permeate through nearly every scientific field, and theoretical and experimental approaches have been developed to connect levels of complexity in other disciplines, e.g., in chemical engineering. Arguably, an analogous breakthrough in microbial ecology poses specific challenges and likely requires its own inventive approaches. Here, we propose a possible roadmap forward that integrates conceptual, experimental, and methodological developments in microbial ecology to address its unique complexity scaling challenges.

## DELINEATING COMPLEXITY

**FIG 1:**
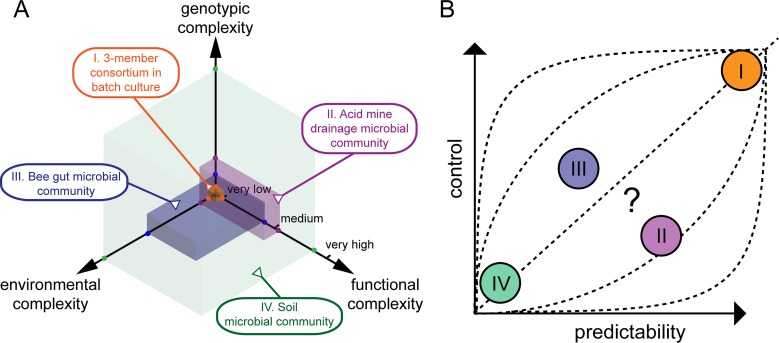
Dimensions of complexity in microbial communities. (A) We define three interdependent axes of complexity. The first spans genotypic diversity and abundance across all domains of life. The second axis encompasses environmental factors at all scales, including habitat structure, physicochemical gradients, and transport processes. The third axis deals with the system characteristics and its emergent properties, ranging from single biochemical reactions to ecosystem functions. All these elements can vary in space and time. Colored volumes exemplify representative types of microbial communities showing various levels of complexity, ranging from very high in all dimensions (soil microbial community) to very low in all dimensions (3-member consortium in batch culture). Other examples are natural microbial systems that show relatively low levels of complexity (microbial communities in the bee gut and from acid mine drainage) and hence represent intermediate research models. (B) Associated with the complexity dimensions are the levels of control and predictability of the microbial system, which are somewhat proportional to the system’s complexity (the relationship between control and predictability, however, may vary with microbial systems and with the type of predicted processes). Complex systems thus tend to be more amenable to descriptive investigations, while simplified ones tend to be more amenable to explanatory investigations. It is of course informative to study complex as well as simple systems. However, there is a trade-off between realism and interpretive power.

First and foremost, we need to systematically describe the various dimensions of complexity present in microbial communities. We suggest three dimensions: genotypic, functional, and environmental, all of which may include spatiotemporal aspects (Fig. 1A). The advantage of this representation is that the level of interdependence between the complexity axes recapitulates fundamental and long-lasting questions in microbial ecology. What is the role of the environment in shaping microbial diversity? How does microbial diversity lead to ecosystem functioning? Moreover, combining the three axes leads to further important questions. For example, how does the spatial arrangement of different genotypes in their environment relate to key community functions? How can community functions be altered, complemented, or restored by introducing nonnative genotypes at certain points in space or time? Of course, some level of subjectivity is inevitable in the choice of dimensions and the meaning of the graduation of the axes. We see this as an opportunity to start a conversation within the community, not as a final word on the matter. Discussing the dimensions of complexity more explicitly would help to suggest precise research questions and invigorate necessary theoretical and methodological discussions in the field.

Equally important is the relationship between complexity, control, and predictability (Fig. 1B). Control refers to how much leeway researchers have on setting the (biological and physicochemical) variables, while predictability indicates how well the behavior of a given microbial system can be anticipated. We suggest that a focus on predictive power would help to guide new experiments and frame results in a meaningful way. Predictability can itself be decomposed into the three complexity dimensions. It may be that emergent community functions can be relatively well predicted based on some environmental factors, while genotypic composition cannot.

## EXPANDING RESEARCH MODELS

Because of the reductionist-holistic divide, most studies are conducted near the “very low” or “very high” mark on a given complexity axis (Fig. 1). Nevertheless, there is today an unprecedented impetus to investigate microbial communities and environments at intermediate levels of complexity and in each of the dimensions or combinations thereof. Recent studies have designed representative bacterial communities of 5 to 20 members (low-to-medium genotypic complexity) from soil, plant, or human gut environments (8–10). New laboratory systems are available that offer more structured environments (low-to-medium environmental complexity), such as artificial soils and porous habitats (11–13), model marine particles (14), or bioreactor granules (15). These systems permit us to address new questions. Does the functional complexity of a microbial community observed with a few genotypes remain the same as incrementally more genotypes are added to the community? If not, at what level of genotypic complexity does functionality no longer increase and why? How does spatial organization influence community functions?

On the other side of the spectrum, holistic approaches could focus on natural microbial communities of lower genotypic, functional, or environmental complexity that are more amenable to experiments and theoretical modeling. Canonical examples include acid mine drainage (16) and insect-associated microbial communities (17). Studies at intermediate levels of complexity offer an opportunity to combine and apply tools across the holistic-reductionist divide. For example, after selecting a natural microbial community of interest, metagenomics could be used to characterize the individual components in detail, while metatranscriptomics could guide the construction of reporter systems to quantify gene expression and infer processes of interest. Subsequently, selected (original or engineered) isolates from the microbial community could be assembled together and the dynamical and emergent properties of the simplified assemblages investigated in controlled environments such as microporous networks. In a feedback loop, mechanisms identified in the simplified assemblages would form the basis of new predictive models whose relevance could be tested on the original community of interest.

## REFINING RULES TO TAME COMPLEXITY

Useful simplifying principles should apply to each of the three interdependent dimensions of complexity. Genotypic complexity may be reduced by identifying and focusing on core members of the community. “Core” may refer to the importance of a genotype either in affecting the ecological and evolutionary dynamics of the ecosystem or in maintaining key functional aspects. If the latter is of greater interest, complexity could be reduced to eliminate functional redundancies and group genotypes into operational functional units defined by the products or processes under study (18). Currently, the functionality dimension is often described separately from the genotypic one in applied scenarios, such as wastewater treatment. Establishing the mapping between the two dimensions is one of the ongoing challenges (see reference 19 for recent progress in linking microbial community structure to function).

Environmental complexity, the third key component, is little understood but needs to be integrated with the other two. Many of the environments of interest are highly stratified with many available niches that are difficult to identify and delineate. Even within each niche, nutrient availability can affect the nature of interspecies interactions (20), carbon sources can determine community structure (21), and the habitat can constrain functional complexity (22). Notably, if genotypes are separated in space, their interactions may be constrained. Knowing the spatial distribution of genotypes could thus allow us to reduce the number of possible interactions drastically: a community of 1,000 genotypes may theoretically result in 5 × 10^5^ possible pairwise interactions, but if populations are heterogeneously distributed across space and if a cell has only a few neighbors, the number of realized interactions may be several orders of magnitude smaller.

For all three dimensions, mathematical models are needed that can bridge the complexity scales, which may help to distill key parameters and describe their interplay. Most modeling efforts have focused on genotypic complexity and interspecies interactions. For example, recent efforts to predict community structure have used pairwise interactions to extrapolate future genotypic complexity with some success (23), and mathematical methods have been proposed to infer interaction types and strengths in complex communities (24). Models focusing on functional and environmental complexity will be key to advancing our understanding.

In sum, communicating a clear representation of microbial complexity and its dimensions and working at their intersection would help to establish formalized methods and rules for complexity solving and thus provide a framework for bridging the holistic-reductionist divide.

We have argued for new principles to overcome the intricacy of natural microbial communities, as well as for an outlook that overpasses the extremes of utmost control and irreducible complexity. While some level of unpredictability will necessarily always be a characteristic of microbial communities and their emergent properties (due to stochastic or chaotic processes), increasing knowledge and computational power may in the long run tilt the scales in favor of reductive explanatory power. In principle, we should be able to conceive predictive equations containing variables such as strain abundance and distribution in space (genotypic complexity), and spatial scales and boundary conditions (environmental complexity), in order to estimate the probability and extent of a set of processes (functional complexity). The challenge is to accurately measure those variables and to place them in a meaningful context. The rapid progress in doing so, which is under way in the field, is a good reason for optimism.
